# Is the macrophyte diversification along the trophic gradient distinct enough for river monitoring?

**DOI:** 10.1007/s10661-016-5710-8

**Published:** 2016-12-03

**Authors:** Krzysztof Szoszkiewicz, Anna Budka, Karol Pietruczuk, Dariusz Kayzer, Daniel Gebler

**Affiliations:** 1 Department of Ecology and Environmental Protection, Poznan University of Life Sciences, Poznań, Poland; 2 Department of Mathematical and Statistical Methods, Poznan University of Life Sciences, Poznań, Poland; 3 Provincial Environmental Inspectorate in Poznan, Poznań, Poland

**Keywords:** Rivers, Macrophytes, Trophy, Freshwater assessment, Ecological status

## Abstract

The variation of a number of parameters characterizing aquatic plant assemblages in rivers across a wide trophic gradient was investigated to evaluate their usefulness for a Polish national river monitoring system. Analyses were conducted at 100 sites included in the national river monitoring system, representing a uniform river type, i.e., small- and medium-sized lowland rivers with a sandy substrate. Results of botanical surveys, which were supplemented with comprehensive monthly quality records, were obtained from the national monitoring database. By analyzing the Jaccard distances of the botanical metrics using the adonis function, the variation in species composition between rivers of different trophic status was determined. The group consisting of the most degraded rivers was the most homogeneous in terms of botanical composition. The cleanest rivers displayed a high level of heterogeneity within their group, as numerous different unique species were found there at low frequencies. The variation of the macrophyte metrics used to assess the ecological status (Macrophyte Index for Rivers (MIR) and River Macrophyte Nutrient Index (RMNI)) reflected a trophic gradient. We confirmed that vegetation diversification along a trophic gradient is evident enough to detect degradation in a five quality class system.

## Introduction

Macrophytes are aquatic plants growing in water which are large enough to be visible with the naked eye and are almost always identifiable in the field. They include higher aquatic plants, vascular cryptograms, and bryophytes, as well as structural macroalgae (Westlake 1975; Holmes et al. 1999). Macrophytes have been extensively studied for decades, and their ecological properties were a frequent topic of research over a long period (Westlake 1975; Wiegleb 1979; Haslam 1982; Haslam 1987).

The development of aquatic plant assemblages strongly depends on a variety of abiotic and biotic factors. It is assumed that the most important of them are nutrient concentrations (Westlake 1975; Robach et al. 1996; Schneider et al. 2000; Thiébaut et al. 2002; Szoszkiewicz et al. 2006; Dodkins et al. 2012), flow velocity (Westlake 1975; Dawson 1988; Fennessy et al. 1994), hydrological conditions (Westlake 1975; Haslam 1987; Baattrup-Pedersen and Riis 1999), pH (Tremp and Kohler 1995), carbonate hardness, shading (Westlake 1975; Dawson and Kern-Hansen 1979), hydromorphological modifications (O’Hare et al. 2006), and landscape pattern (Wiegleb et al. 2015). A significant and apparent response of vegetation provides a useful indication of persistent and long-term habitat changes in aquatic ecosystems, which have been widely used as indicators of water quality in streams and rivers for many decades (Wiegleb 1979; Haslam 1982; Holmes et al. 1999; Ceschin et al. 2010). Nowadays, this group of organisms is an obligatory element in the monitoring of the ecological status of surface waters in EU countries under the Water Framework Directive (WFD, European Commission 2000). For river monitoring purposes, several systems based on aquatic plants have been developed, and some of them have been integrated into national monitoring programs, e.g., in the UK (Willby et al. 2009), France (Haury et al. 2006), Germany (Schaumburg et al. 2004) and Poland (Szoszkiewicz et al. 2010).

The implementation of macrophytes, as well as other biological groups, as elements of freshwater monitoring, was a great success on the part of ecologists, resulting from decades of studies on environment–macrophyte interactions. Nowadays, after almost 10 years of biological monitoring, several problems still exist in the interpretation of the signals delivered by aquatic organisms (Hering et al. 2010). Some skeptical arguments also concerned the idea of macrophyte indication (Demars and Edwards 2009; Demars et al. 2012a; Wiegleb et al. 2014a), in view of the difficulty of statistical detection of important ecological gradients, particularly trophy (as well as altitude and alkalinity), due to collinearity of ecological gradients as well as insufficiency of databases to estimate spatial and temporal variation of macrophyte metrics. Moreover, Wiegleb et al. (2015) raised questions on the idea that a classification-independent single metrics method can yield reasonable results under complex conditions. By means of stratification of sampling or classification of rivers, better results are achieved.

We tried to challenge the above difficulties, as we based our research on the national river monitoring database which provides comprehensive information on the physicochemical status of waters. The analyzed database enabled us to consider a very wide trophic gradient within the selected homogeneous river type. In this way, our investigation is exceptional in relation to other similar studies both in terms of the width of the trophic gradient and the comprehensiveness of the physicochemical determinations, as well as typological homogeneity, taking into account substrate, catchment size, altitude, and level of hydromorphological degradation (Szoszkiewicz et al. 2006; Demars and Edwards 2009; Birk and Willby 2010; Ceschin et al. 2010; Hering et al. 2010; Wiegleb et al. 2014a).

Our study aimed to identify the variation of different macrophyte metrics along a trophic gradient and to verify the taxonomic distinctness of macrophyte communities developing in waters with different levels of eutrophication. Moreover, we aimed to assess the variability of botanical metrics in relation to their use in the classification of rivers. We hypothesize that vegetation is diversified along a trophic gradient in such a way that we can discriminate distinct plant communities representing five quality classes.

## Materials and methods

### Site selection

**Fig. 1:**
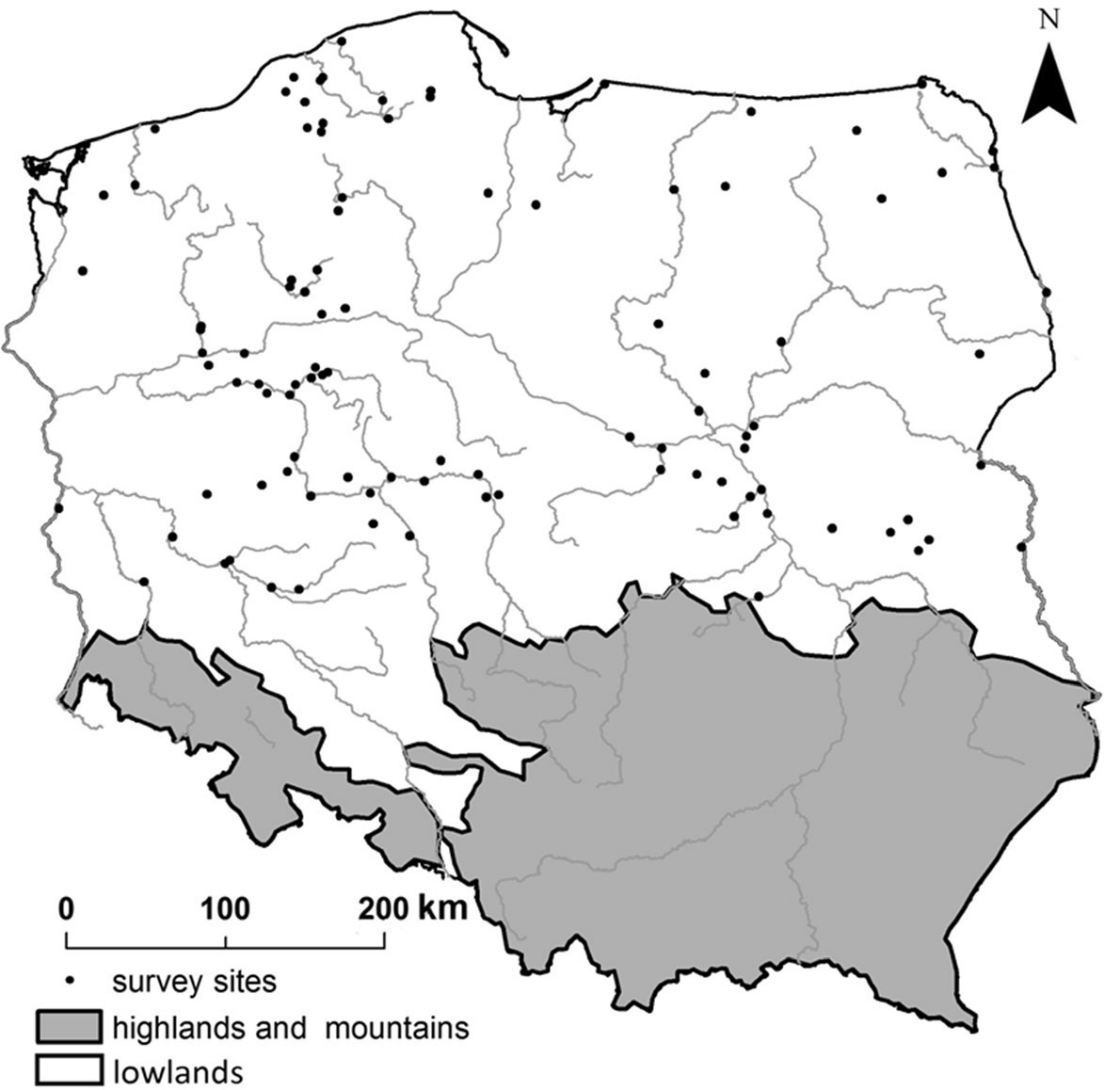
Location of selected experimental sites

Analyses were conducted throughout Poland at 100 river sites included in the state environmental monitoring survey system. Initially, 338 sites were pre-selected from the monitoring database, which were all classified under a single abiotic type—sandy lowland rivers (Fig. 1). In terms of altitude, all the rivers were located below 200 m a.s.l., while in terms of catchment size, they were smaller than 1000 km^2^ (small and medium rivers, according to the WFD). Only rivers with substrate predominantly composed of sand were included in the study. Artificial canals and rivers which were strongly hydromorphologically transformed were excluded from the analysis.

At the next stage of selection, physicochemical criteria were considered based on the state environmental monitoring database, where water samples were collected at monthly intervals (12 monthly samples). The selected sites represent a wide trophic gradient based on the concentration of phosphorus (reactive and total phosphorus) and nitrogen (total nitrogen). Only sites at which nitrogen concentration correlated with the concentration of phosphorus were selected for analysis. The site selection process was also supported by several statistical methods which are described in detail below.

Under the site selection process, we chose from the national monitoring database 20 of the pristine watercourses and 20 of those exhibiting the most advanced eutrophication. Moreover, 60 mesotrophic sites with an average concentration of nutrients were also included. The k-means clustering based on water physicochemical parameters allowed the distinction of the survey sites into the three mesotrophic classes. In this way, 100 selected sites were divided into 5 groups corresponding to a gradient of eutrophication from the least eutrophic river sites (river trophic class 1) to those with advanced eutrophication (class 5).

### Macrophyte surveys

Macrophyte surveys were carried out on the selected 100 sites in the years 2008–2011, during the summer season between July and early September. The macrophyte surveys were undertaken using the Polish national monitoring method based on the Macrophyte Index for Rivers (MIR) (Szoszkiewicz et al. 2010), which was developed to meet the requirements of the Water Framework Directive. The field procedure of the MIR method corresponds to most other European macrophyte methods, and it was intercalibrated with other European macrophyte assessment methods (Birk and Willby 2010). The survey reach was 100 m in length, where all submerged, free-floating, amphibious, and emergent monocotyledonous and dicotyledonous plants, as well as filamentous algae, liverworts, mosses, and pteridophytes, were identified. The assessment also included macrophytes attached or rooted in parts of the river bank that are likely to be submerged for more than 85% of the year. The cover of each species was recorded using the following nine-point scale: <0.1, 0.1–1, 1–2.5, 2.5–5, 5–10, 10–25, 25–50, 50–75, and >75%. A glass-bottomed bucket was used to aid observations. For non-wadeable parts of the largest rivers, a grapnel was used to retrieve macrophyte from the channel.

### Statistical analysis

Principal component analysis (PCA) was used to analyze the physicochemical monitoring data in the site selection process. PCA made it possible to visualize the distribution of selected sites in the multivariate physicochemical matrix, and we were able to select 100 sites following the trophic gradient and classify them in five groups representing different trophic levels.

To confirm the qualitative distinctness of identified groups of rivers representing different water trophic levels, canonical variate analysis (CVA) was performed in relation to physicochemical parameters. This method consists in the transformation of the analyzed matrix into a set of new variables, which carry similar information but are distributed in a multivariate Euclidean space (Lejeune and Caliński 2000). Elements of the CVA matrices included differences between mean values for nine physicochemical indices in various river quality classes and means for five trophic classes.

Botanical data collected in the field allowed us to calculate the mean Jaccard index (Jaccard 1912) for groups of rivers representing different trophic classes. When analyzing the Jaccard index, we used the *A* value, the mean value of the Jaccard index, to estimate similarities within the considered trophic groups of rivers, and the *B* value, which is the mean value of the index between groups. In addition, we used the CS value, denoting the classification strength (CS = *A* − *B*). The CS value estimates whether the similarity of species composition of the surveyed sites is greater within or between river types (Digby and Kempton 1987; Mielke and Berry 2001; Warton et al. 2012).

The significance of differences was tested with a permutation test using the adonis procedure (analysis of variance using distance matrices) utilizing a permutation test with the pseudo-*F* statistic (Zapala and Schork 2006). Typical uses of the adonis function include the analysis of ecological community data (distance matrices for samples of species) (e.g., Zapala and Schork 2006). The adonis function is an alternative to AMOVA (nested analysis of molecular variance; Excoffier et al. 1992) for both crossed and nested factors. The similarities and differences in species composition between rivers representing five quality classes were demonstrated according to the geombinatoric approach using Venn diagrams (Ruskey et al. 2006).

Based on the macrophyte database, several macrophyte metrics were calculated, including basic diversity metrics such as species richness, the Shannon diversity index (Shannon and Weaver 1949), the Simpson diversity index (Simpson 1949), detectable diversity (the inverse Simpson index), and evenness (Pielou 1966). Moreover, the percentage of the river bottom area covered by macrophytes (total cover) was estimated. Finally, two metrics indicating the ecological status of rivers were calculated, namely the Macrophyte Index for Rivers (MIR) (Szoszkiewicz et al. 2010) and the River Macrophyte Nutrient Index (RMNI) (Willby et al. 2009). The consistency and comparability of the classification results delivered by both methods were tested within the EU Water Framework Directive intercalibration exercise (Birk and Willby 2010).

Variation in botanical metrics between trophic classes of rivers was analyzed using the Kruskal–Wallis test, because the assumptions for analysis of variance (ANOVA) were not met and the character of the variables prevented the application of ANOVA (Hollander and Wolfe 1973). When the null hypothesis asserting a lack of differences between mean indices for individual trophic classes was rejected, multiple comparisons (post hoc tests) were completed (Siegel and Castellan 1988).

Statistical analyses were performed using the R computational platform. The available packages, i.e., “vegan” v.2.2–0 (Oksanen et al. 2013), “agricole” v.1.2–0, “car” v.2.0–22, “devtols” v.1.7.0, “ggplot2” v.1.0.0, “gplots” v. 2.14.1, “graphics” v.3.1.1, “pgirmess” v. 1.5.9, and “prabclus” v. 2.2–4, were used.

## Results

**Fig. 2:**
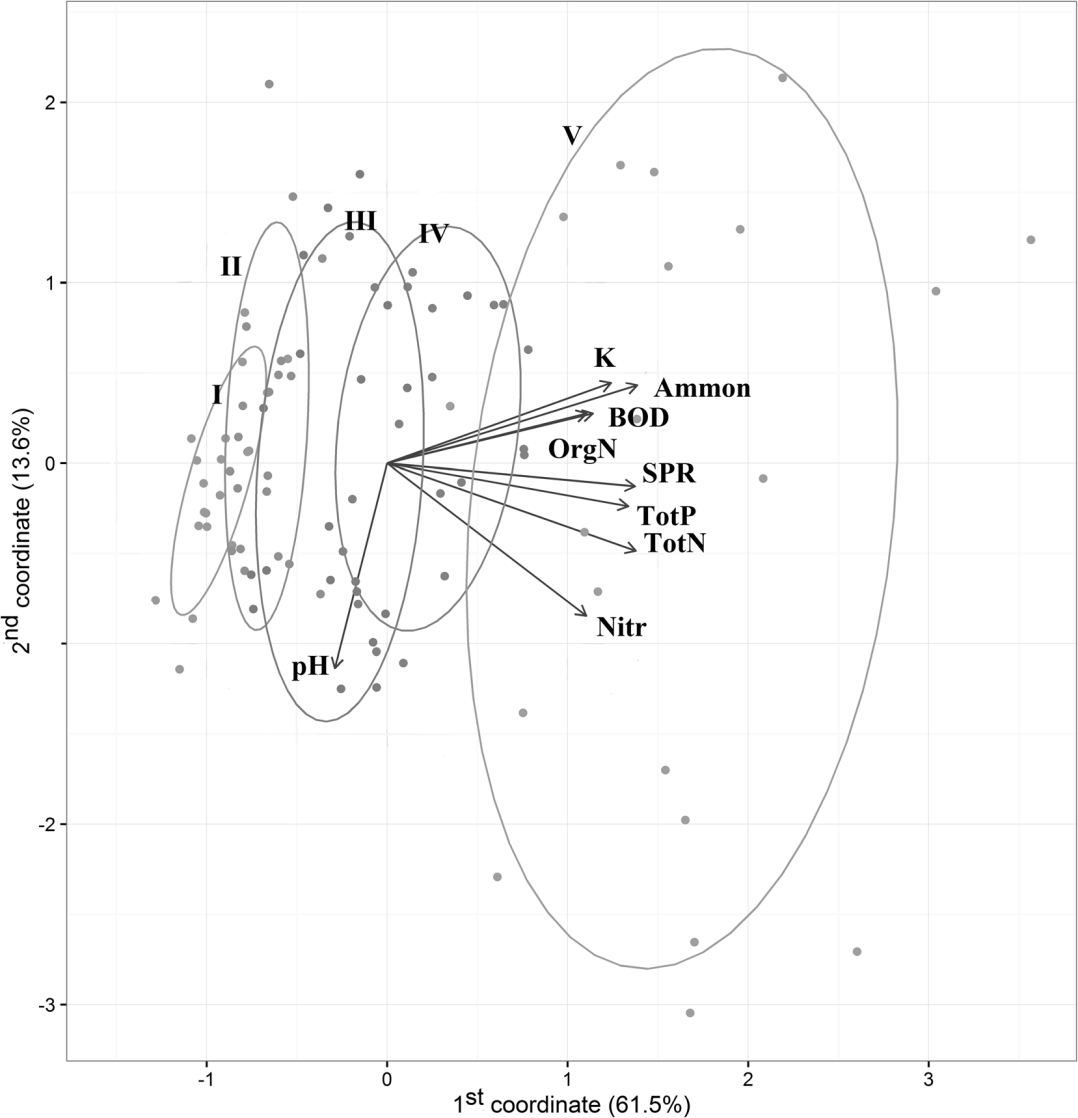
Principal component analysis of the physicochemical matrix. Ellipsoids indicate 67% confidence

The PCA analysis indicated that the considered physicochemical matrix contains a wide trophic gradient represented by almost parallel total phosphorous and total nitrogen plots (Fig. 2). Other parameters in general followed this gradient, most strongly for reactive phosphorous and slightly less for nitrate, ammonia, organic nitrogen, Kjeldahl nitrogen, and biochemical oxygen demand (BOD). Moreover, PCA analysis made it possible to visualize the distribution of selected sites in the multivariate physicochemical matrix.

The investigated rivers represented a very wide gradient of hydrochemical quality. The five identified trophic classes showed a broad range in terms of the concentrations of most water parameters (Table 1). This concerned key indicators of the trophic status of water, i.e., different forms of nitrogen and phosphorus, as well as conductivity and BOD_5_. An exception in this respect was pH, which showed no variation between the identified groups.

**Table 1:** Descriptive statistics of the physicochemical variables of rivers representing five trophic classes

Trophy class	Variable	Ammonium	Nitrate	Kjeldahl N	Total N	Organic N	BOD	Total phosphate	Ortho-phosphate	pH
		(mg N-NH_4_/l)	(mg N-NO_3_/l)	(mg N/l)	(mg N/l)	(mg N/l)	(mg O_2_/l)	(mg P/l)	(mg PO_4_/l)	–
I	Mean	0.10	0.41	0.93	1.35	0.83	2.37	0.209	0.111	7.97
	Median	0.06	0.35	0.91	1.36	0.78	2.22	0.215	0.120	7.95
	Variance	0.01	0.05	0.08	0.16	0.07	0.45	0.004	0.003	0.02
II	Mean	0.16	1.15	1.13	2.30	0.97	2.33	0.401	0.193	7.79
	Median	0.10	1.07	1.13	2.27	0.95	2.19	0.405	0.201	7.82
	Variance	0.02	0.53	0.09	0.50	0.10	0.41	0.002	0.005	0.05
III	Mean	0.26	2.41	1.48	3.93	1.22	2.47	0.570	0.344	7.79
	Median	0.22	2.39	1.43	4.06	1.21	2.50	0.563	0.328	7.81
	Variance	0.03	1.43	0.24	1.79	0.24	0.32	0.006	0.005	0.05
IV	Mean	0.48	2.53	1.91	4.44	1.43	3.75	0.868	0.539	7.78
	Median	0.42	2.37	1.93	4.51	1.34	3.97	0.851	0.531	7.82
	Variance	0.09	1.31	0.24	1.57	0.22	1.06	0.017	0.003	0.05
V	Mean	1.50	5.27	3.35	8.60	1.84	4.40	1.718	1.150	7.80
	Median	1.09	4.57	2.91	8.56	1.52	4.48	1.682	1.139	7.79
	Variance	1.39	10.64	2.26	10.94	0.45	1.43	0.238	0.143	0.04

**Fig. 3:**
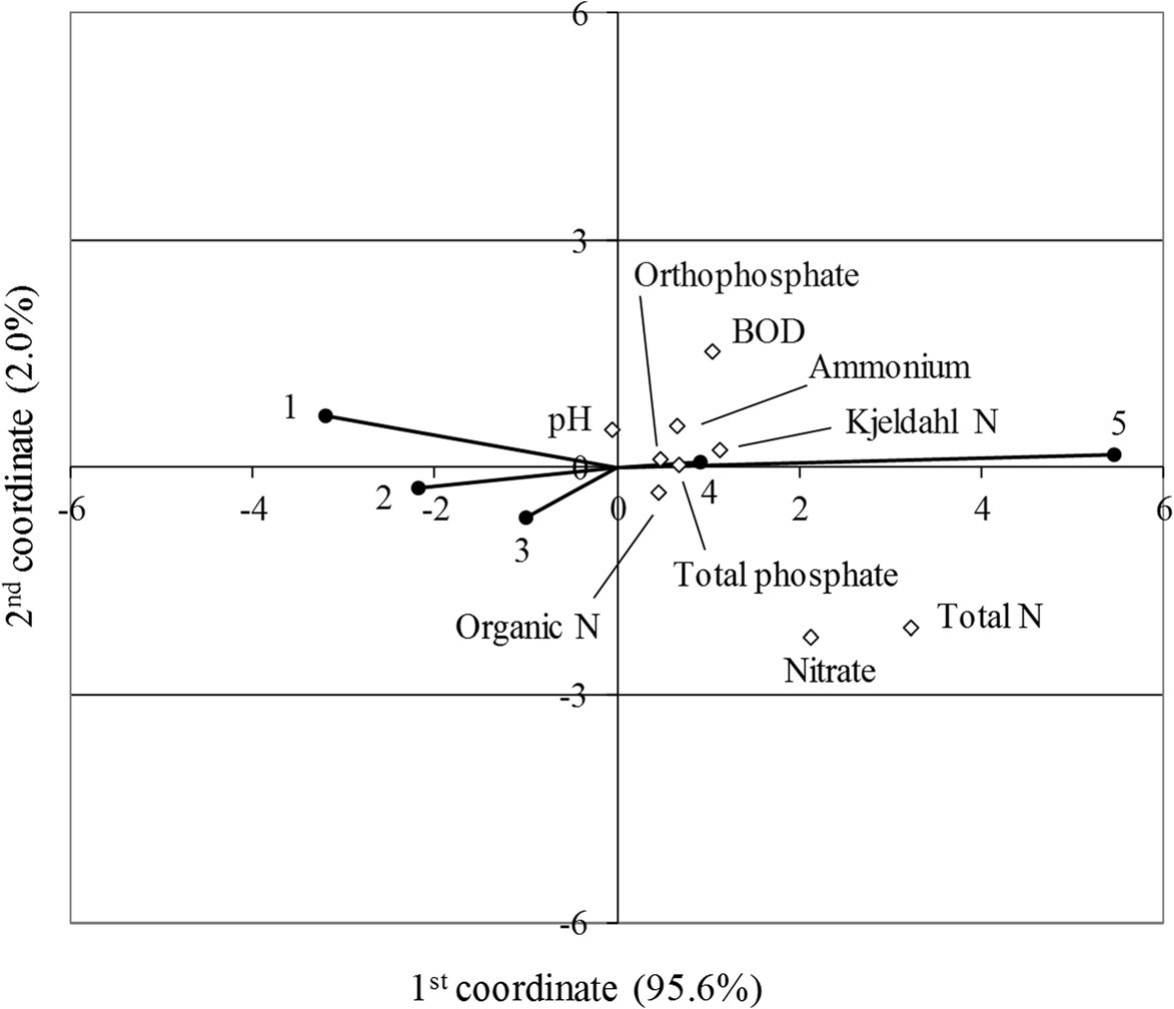
Canonical variate analysis showing the relationship between trophic classes and hydrochemical parameters

The CVA procedure, analyzing yearly mean values of hydrochemical parameters in different river trophic classes and considering their effects in the space of canonical variables, confirmed the qualitative distinctness of the identified river groups (Fig. 3). It was found that the most differentiating factors for the position of individual classes were total nitrogen and nitrate. These parameters operated in parallel with other factors indicating eutrophication. The identified trophic gradient was well reflected by the order of trophic classes, confirming the relevance of the approach for a classification of sites into five quality classes.

Additionally, it was observed that rivers representing trophic class V differed to the greatest extent from the others in terms of physicochemical parameters. This indicates that the group of the most degraded sites was particularly distinct from the other groups as regard to water quality characteristics.

Water pH, which is mostly a proxy of alkalinity, represents an independent direction of variation. It was found that this gradient had no effect on the distribution of points illustrating the position of river trophic classes.

**Fig. 4:**
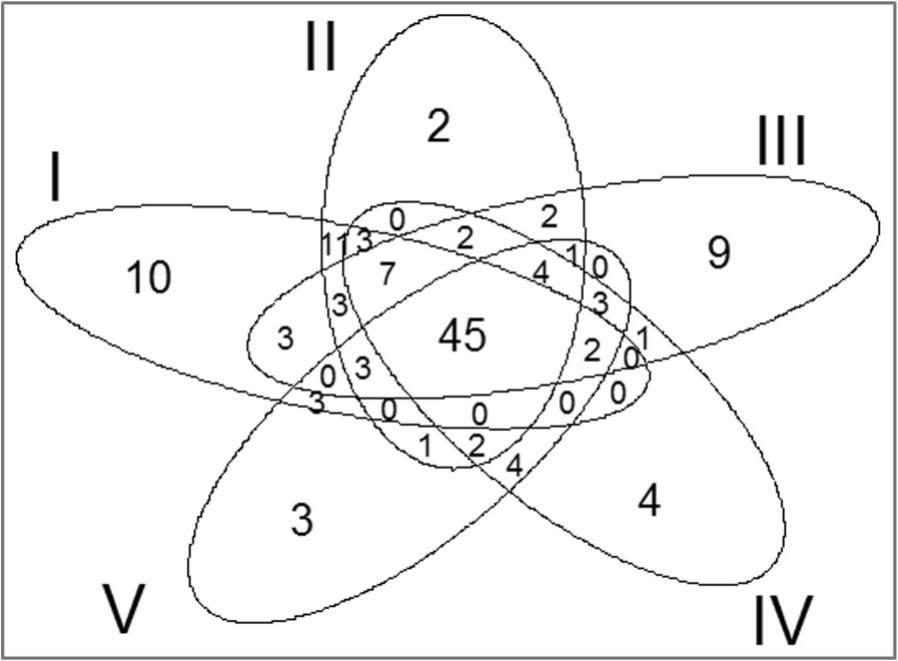
Venn diagram—analysis of the taxonomic differentiation of the five trophic classes (*I*–*V*). *Numbers* show the distribution of shared species

**Fig. 5:**
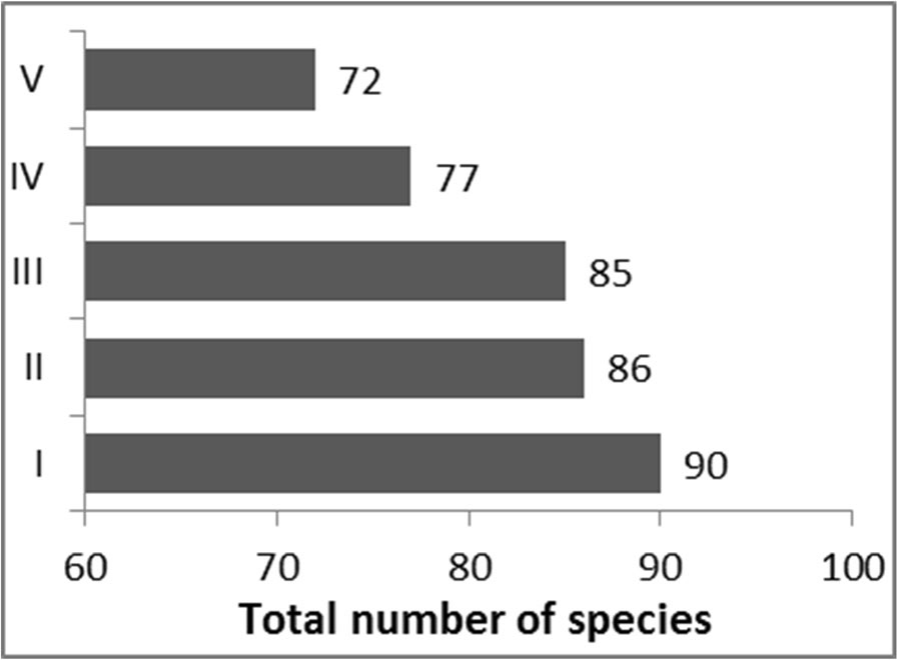
Total number of species within 20 sites representing each trophic class

Calculation of the matrix of Jaccard index means made it possible to analyze variation in species composition in rivers representing different trophic statuses. To support interpretation of the Jaccard index matrix, a Venn diagram was used (Fig. 4). The diagram identifies plant species shared in the rivers representing the complete trophic gradient (45), as well as characteristic species found only at sites corresponding to a specific trophic level. Class II is not well defined (only 2 exclusive species), but 11 species are shared with class I. The total number of species varied among trophic classes, decreasing with increasing degree of degradation (Fig. 5).

The variation of macrophyte communities between river trophic classes was indicated by the positive value of the Jaccard index classification strength (CS = 0.026). Homogeneity of species composition was greater within types (*A* = 0.210) than between types (*B* = 0.184). The mean values of the Jaccard index for rivers representing the respective trophic classes were 0.160, 0.199, 0.218, 0.229, and 0.240 (Table 2). These results showed that the highest degree of heterogeneity (the highest heterogeneity within groups) occurred for the most pristine rivers (trophic class I). This is largely the result of their having the highest total pool of species (90 taxa; Fig. 5). Moreover, the cleanest rivers featured many less common species, whose frequency was low (e.g., *Hippuris vulgaris*, *Cratoneuron filicinum*, *Menyanthes trifoliata*, *Lysimachia thyrsiflora*, *Viola palustris*, *Carex diandra*, *Carex pseudocyperus*). These species were not found in more degraded watercourses; thus, the group of the cleanest rivers exhibited (apart from the lowest intragroup homogeneity) a higher degree of distinctness compared with degraded rivers.

**Table 2:** Average Jaccard index values for different river types

I	0.160				
II	0.170	0.199			
III	0.177	0.203	0.218		
IV	0.156	0.203	0.211	0.229	
V	0.143	0.193	0.200	0.237	0.240
Trophy class	I	II	III	IV	V

With the deterioration of water quality, the value of the Jaccard index increased, which indicates increasing intragroup taxonomic homogeneity. This results from the successively decreasing number of recorded taxa with deterioration of water quality. The total pool of species identified for rivers in the first class was 90, whereas for the more degraded classes, it was 86, 85, 77, and 72, respectively. Moreover, in rivers with the most advanced eutrophication, the number of exclusive species (not recorded in other rivers) was the lowest—there were only three such species (*Juncus articulatus*, *Polygonum nodosum*, *Stigeoclonium* sp.). As a result, the group of the most eutrophic rivers exhibited (apart from the highest intragroup homogeneity) the lowest degree of distinctness compared with rivers exposed to lower water degradation.

The significance of the observed taxonomic variation between rivers representing different trophic classes was tested with the adonis function for analysis of similarity (999 permutations) with the Jaccard matrix of distance. The value of the test statistic in the adonis procedure, the pseudo-*F* statistic, was 0.009. The probability value *p* = 0.016 indicated that species variation between the identified trophic classes of rivers was statistically significant.

**Fig. 6:**
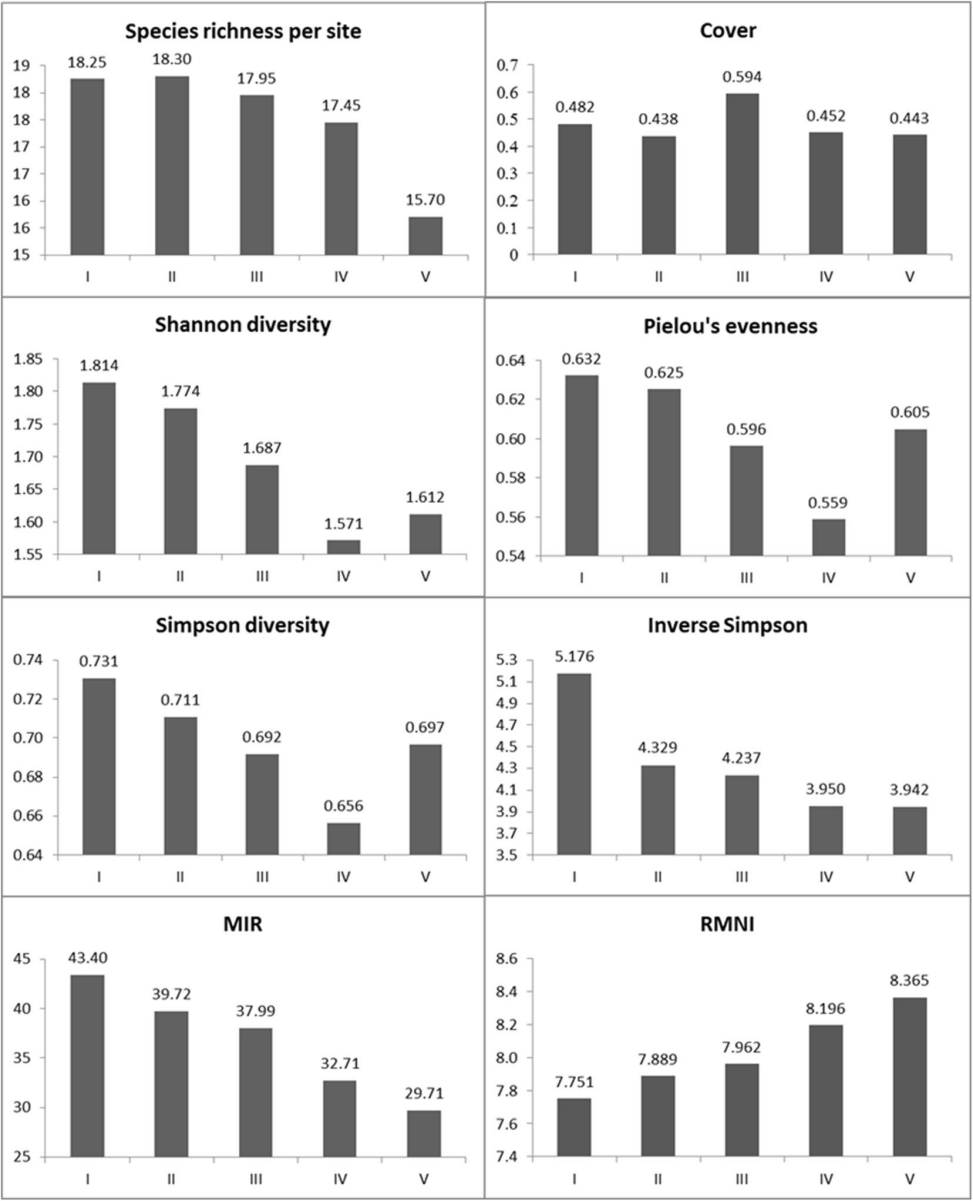
Variability of botanical metrics calculated for different river quality classes

Analysis of variance for botanical diversity metrics, such as species richness, the Shannon index, the Simpson indices (standard and inverse), and evenness, generally showed that less degraded rivers exhibit greater compositional variation than eutrophic rivers (Fig. 6). This refers to parameters connected with both the number of species (species richness) and relative numbers of individual taxa (the evenness index), as well as to indices derived from those parameters (the Shannon and Simpson indices).

Differentiation of mean diversity metrics between rivers representing various trophic classes was detected, but it was not statistically significant according to the Kruskal–Wallis test (Table 3). Mean values of the diversity metrics were associated with a very high intragroup variance. It was found that a large pool of species can be detected most often in a river that is poor in nutrients but sometimes also in degraded watercourses.

**Table 3:** Kruskal–Wallis test for variation in botanical indices between river quality classes

Index	*χ* ^2^	*p* value
Species richness (*S*)	2.49	0.65
Cover	0.99	0.91
Shannon	3.58	0.47
Pielou’s evenness (*J*)	2.63	0.62
Simpson	3.42	0.49
Inverse Simpson	3.42	0.49
MIR	63.00	<0.01
RMNI	33.53	<0.01

It was also shown that greater plant diversity of clean rivers compared to polluted rivers is detectable only with distinct differentiation of the degradation gradient, i.e., between the oligotrophic rivers and those with very advanced eutrophication. Trends of macrophyte diversity change along a trophic gradient were not always proportional; for example, mean species richness of trophic class II rivers was higher than average species number in the top quality rivers. Likewise, in the case of evenness, the most degraded rivers showed a more uniform relative abundance of species than rivers in the third and fourth classes.

In the case of the macrophyte indices used to evaluate the ecological status of rivers (MIR and RMNI), a strong dependence on the trophic status was demonstrated. The statistical test (Kruskal–Wallis) confirmed the significance of differences between the identified trophic classes and the mean MIR and RMNI values (Table 3).

## Discussion

The data used were appropriate for analysis of the response of macrophytes to river degradation caused by eutrophication. A particularly significant part of the analyzed data is the physicochemical dataset where samples were taken monthly during the whole year, for each river site. The quality of our physicochemical data is exceptional within the field of research into aquatic plant ecology, since most studies are typically based on data from a single water sampling (Hering et al. 2006; Szoszkiewicz et al. 2006; Dodkins et al. 2012; Chappuis et al. 2014; Steffen et al. 2014; Jusik et al. 2015) or several samplings at most (Thiébaut et al. 2002; Birk and Willby 2010). Analyses of large scale macrophyte development in relation to comprehensive hydrochemical data obtained from environmental monitoring were carried out by Marzin et al. (2012), but the typological differentiation was not respected in their analysis. In the study by Wiegleb et al. (2015), the gradient was only three classes long (comparable to classes II–IV in this paper), and thus, many assessment methods did not work well.

Our database of physical and chemical parameters was shown to represent a very wide range of trophy, but the existing gradient of nutrient concentrations among Polish rivers is even wider. The national monitoring system focuses on the major and representative watercourses, and most of the pristine rivers were absent. The range of reference rivers was confirmed for Poland by Jusik et al. (2015), and these are not included in the national monitoring system. The situation is similar with heavily degraded sites, which have been detected locally and are not monitored at the national level. The detected relationships between macrophytes and trophy appear already to be confirmed statistically, but the potential impact of trophic gradient is even more evident within the complete existing gradient in lowland rivers.

A great advantage of this study was the extent of the analyzed trophic gradient. The five identified trophic classes showed considerable variation in terms of concentrations of various water parameters. This concerns key indicators of the trophic status of water, i.e., different forms of nitrogen and phosphorus, as well as pH and BOD_5_. The nutrient concentration in the high-quality rivers was very low: the mean annual concentration of phosphates was 0.111 mg PO_4_/l, while that of nitrate nitrogen was 0.409 mg N-NO_3_/l. In comparison with, e.g., Neal et al. (2006), these are very low concentrations, which, according to Pardo et al. (2012), can be regarded as pristine reference levels. Similarly, the most degraded of the investigated rivers had a mean concentration of phosphates of 1.150 mg PO_4_/l and a mean concentration of nitrate nitrogen of 5.271 mg N-NO_3_/l. Comparing these results with, e.g., Muylaert et al. (2009) and Howden et al. (2009), we can conclude that they represent a very high level of nutrients, indicating that an extreme stage of eutrophication has already been reached (Wetzel 2001).

We confirmed the hypothesis that vegetation is diversified along a trophic gradient and that we can discriminate distinct plant communities representing five quality classes. Calculation of the Jaccard index enabled an analysis of species variation in rivers of different trophic status. Variation in macrophyte communities between trophic classes was indicated by the positive value of the classification strength, and this was the result of the greater species homogeneity within trophic groups than between them. The effect of water quality, including nutrient concentration, on the development of vegetation in aquatic ecosystems has also been confirmed by other studies (e.g., Manolaki and Papastergiadou 2013; Chappuis et al. 2014; Steffen et al. 2014).

The highest degree of homogeneity (the highest intragroup homogeneity) was found among the most degraded rivers (fifth trophic class). This results from the successively decreasing mean number of species recorded with deterioration of water quality. Strongly degraded rivers were very uniform in terms of their taxonomy and contained only small numbers of exclusive species (not recorded in other rivers). The decrease in plant diversity, as well as overrepresentation of ecologically tolerant species, in strongly impacted sites has been confirmed in other studies (Schaumburg et al. 2004; Kuhar et al. 2011; Steffen et al. 2014).

On the other hand, the high-quality sites exhibited low values of the Jaccard index, which indicates a lower taxonomic intragroup homogeneity. In the cleanest rivers, several unique species were recorded, whose frequency was low. It is remarkable in the context of biodiversity conservation—our results proved that high-quality rivers play an important role in maintaining the biodiversity of aquatic ecosystems. Moreover, this finding confirms the importance of rare species of macrophyte indication in rivers. These are often removed from data analysis for the sake of convenience.

These species were not found in more degraded watercourses, and therefore, the group of the cleanest rivers exhibited (apart from the lowest intragroup homogeneity) the greatest degree of distinctness in comparison with the rivers classified in groups of inferior quality. The occurrence of many rare species in clean waters has already been reported in the literature (Schaumburg et al. 2004; Steffen et al. 2014). In turn, the disappearance of rare sensitive taxa in watercourses with increased nutrient content was described by Dodkins et al. (2012), who investigated lowland rivers in the UK. Changes in macrophyte species and their population size under the influence of water habitat disturbance have also been reported by other authors (e.g., Kohler 1975; Steffen et al. 2013, 2014; Wiegleb et al. 2015).

Our analysis proved that the vegetation of polluted rivers is poor in species and is dominated by common species, whereas the flora of pristine watercourses is relatively diverse and rich in rare species. Moreover, the floral homogeneity of the river sites increases with increasing eutrophication. It is probably due to the high importance of habitat variation among unspoiled sites. Unpolluted rivers more distinctly reflect geographical and geological factors. We analyzed a uniform river type, but the habitat could still be influenced by local young and old glacial deposits, alluvial sands, limestone rock, and peat bogs (Wiegleb et al. 2015).

Analysis of the macrophyte diversity metrics (species richness, the Shannon and Simpson indices, the evenness index) confirmed the threats to biodiversity caused by river eutrophication. Diversity metrics attained higher values in pristine sites compared with those impacted by a high level of nutrients. The diversity value of unpolluted aquatic ecosystems has already been reported for plants (e.g., Jeppesen et al. 2000; Fabris et al. 2009; Jusik et al. 2015) and other aquatic organisms, such as benthic invertebrates and zooplankton (Petchey et al. 2004; Jeppesen et al. 2000). This applies to parameters connected with both the number of species (*N*) and their relative abundance (*J*), as well as to indices derived from these parameters (*H*, *D*). The variation in diversity indices was strongly differentiated within the identified trophic classes—for each of these groups, variance in the indices of botanical compositional variation was very high, i.e., localities rich in species may be found both among sites with water that is poor in nutrients and among those that are hydrochemically degraded. As a result, the observed variation between groups of rivers representing different trophic statuses was not statistically significant. A decline of the number of species was observed for lake macrophytes with increasing total phosphorous but mainly in relation to submerged plants (Jeppesen et al. 2000). This was also found in our studies in rivers, where the loss of species richness was more visible for submerged plants than for floating-leaved and emerged macrophytes (Steffen et al. 2013), whereas Demars et al. (2014) and Wiegleb et al. (2014b) observed fluctuation between different growth form types.

Another important finding was the lack of relationship between the macrophyte compositional variation and the trophic gradient, as detected for most of the diversity metrics. In accordance with the intermediate disturbance hypothesis (Connel 1978), the greatest compositional variation is observed at a moderate degree of degradation. It is a pattern found for various organism groups in aquatic habitats, for instance, floating-leaved macrophytes as well as fish and phytoplankton, where species richness was unimodally related to total phosphorus in lakes, all peaking at 0.1–0.4 mg P l^−1^ (Jeppesen et al. 2000). Therefore, higher values of diversity indices of oligotrophic rivers than eutrophic rivers usually refer only to watercourses representing a very wide degree of degradation (between pristine and heavily degraded sites).

As a result of the high variance and the lack of dependence between the degree of water degradation and the compositional variation, the applicability of botanical diversity indices in monitoring is very limited. Problems with the application of diversity indices in the assessment of river quality were also reported by Thiébaut et al. (2002), who investigated rivers in the Northern Vosges (northeast France).

We confirmed that the MIR index reflects the degree of river degradation well. The differences between the mean MIR values of river quality groups representing a wide trophic gradient were obvious and significant. This represents the expected distinctiveness of the MIR index, which was developed for the requirements of WFD and was calibrated to indicate strictly the trophic factor in flowing waters (Szoszkiewicz et al. 2010). The design of MIR was relatively simple when compared with some other metrics (e.g., Ali et al. 1999; Dodkins et al. 2005; Willby et al. 2009), so as to avoid a situation where the index is so complicated that it is difficult to identify the ecological significance of the final results (Demars, Potts, et al. 2012b). The British metric for ecological quality assessment, RMNI, showed similar attributes to MIR in reflecting the trophic gradient. Similarly to several other studies (e.g., Thiébaut et al. 2002; Birk and Willby 2010), our analyses demonstrated the applicability of macrophyte methods for ecological quality assessment in relation to trophic degradation.

Analysis of macrophyte indices revealed that our second hypothesis, in which macrophyte metric differentiation in the trophic gradient based on hydrochemical parameters can be reflected by the five class quality scale, was confirmed. However, trends of macrophyte diversity change along the trophic gradient were not proportional, but the variation of the metrics used to assess the ecological status (MIR and RMNI) reflects the physicochemical gradient. This relationship was almost proportional and statistically significant. These two metrics, like other metrics developed for the purpose of ecological status assessment in Europe (e.g., IBMR, Haury et al. 2006; RI, Schaumburg et al. 2004) utilized in their local conditions (EU countries), can be recommended for monitoring and can be applied for ecological classification of rivers which are degraded by eutrophication.

We proved that the macrophyte metrics may reflect the degree of river trophic degradation, since we confirmed the relationship between quality classes based on physicochemical parameters and macrophyte-derived ecological classification. We used quality class comparisons to avoid regression due to non-linear relationships, collinearity, and interaction effects. By basing the analysis on classification, the noise was reduced, and thus, the underlying pattern (gradient) could be identified. Regression analysis often fails to reflect the relationship between plants and environmental factors in rivers (Demars, Potts, et al. 2012b). Searching for linear relationships between ecological indexes and a single physical or chemical parameter seems to be a too simple way to describe relationships of the river ecosystem. An ecosystem is a complex set of relationships among living resources, habitats, and residents of an area, hence requiring a more advanced analytical approach (Mooij et al. 2010, Szoszkiewicz et al. 2014). Our analytical approach was also quite simple, but by basing it on classification, we attempted to avoid direct assumptions about the proportionality of water quality variables and macrophyte assemblage metrics. The analysis by groups (comparison between two kinds of quality classification) was largely resistant to non-linear relationships and interactions between dataset variables.

We support the idea that river habitat–macrophyte interactions can be limited to a uniform model or technical linking group of models (Mooij et al. 2010). In our case, as mentioned above, the ecological pattern was identified by a classification approach. Moreover, we agree that applying multiple modeling approaches concurrently, using existing models and model components (including trophic status), can help to develop an integrative scientific approach to the functioning of river ecosystems and to provide managers with essential ecological information for water quality management (Mooij et al. 2010). The response of macrophytes to the set of physicochemical variables can be reflected using neural networks, which were also successfully applied to similar questions by Wiegleb et al. (2014b) and Gebler et al. (2014).

In our study, we were able to evaluate the habitat–macrophyte interactions in typologically homogeneous conditions. According to the principles of ecological classification, the results of environmental assessments should be compared with the reference conditions corresponding to the given type of water. Within uniform water types, variation of non-anthropogenic factors is slight and certainly does not exceed the broad gradient of anthropogenic degradation. We do not have information from the available literature on any uniform type of river subjected to human pressure where the gradient of pH was so great as to exceed the strong trophic factor unless it is only a proxy of the alkalinity gradient. The river system analyzed by Demars et al. (2012a) involved the rivers of northeastern France, which may actually be characterized by a wide gradient, more significant for macrophytes than trophy, but various types are represented within these rivers.

Another important advantage of the use of macrophytes in monitoring is the high reliability of indication, while variability of chemical and physical water analysis is often very high (Macdonald et al. 1995; Brunet and Astin 1999; Bowes et al. 2003; Howden et al. 2009; Muylaert et al. 2009; Zieliński and Jekatierynczuk-Rudczyk 2014; Zieliński et al. 2009). Temporal variance of macrophyte metrics can be sometimes considerable even without change in abiotic parameters (Wiegleb et al. 2015) but generally is low enough for the time scale of 3 years, as required in WFD monitoring (Staniszewski et al. 2006, Demars et al. 2014).

Nutrient concentrations in rivers are highly variable and show no significant trend in many catchments (Macdonald et al. 1995). Nutrient concentrations in surface waters may fall within a very wide range of values over several years (Brunet and Astin 1999; Bowes et al. 2003; Howden et al. 2009), within 1 year (Macdonald et al. 1995; Zieliński and Jekatierynczuk-Rudczyk 2014), or even over several months (Macdonald et al. 1995; Sojka et al. 2008), and this high variance has also been observed in comparisons of monthly averages (Muylaert et al. 2009; Zieliński et al. 2009). Even a single rainfall event may significantly affect physical and chemical characteristics of the river water (House and Warwick 1998). The high degree of variability of the assessment of physicochemical parameters is certainly an important reason for the low rates of correlation between nutrient pressures and biological metrics (Hering et al. 2006; Demars and Edwards 2009; Demars, Potts, et al. 2012b; Chappuis et al. 2014; Steffen et al. 2014).
